# Ebolavirus Species-Specific Interferon Antagonism Mediated by VP24

**DOI:** 10.3390/v15051075

**Published:** 2023-04-28

**Authors:** Palaniappan Ramanathan, Bersabeh Tigabu, Rodrigo I. Santos, Philipp A. Ilinykh, Natalia Kuzmina, Olivia A. Vogel, Naveen Thakur, Hamza Ahmed, Chao Wu, Gaya K. Amarasinghe, Christopher F. Basler, Alexander Bukreyev

**Affiliations:** 1 Department of Pathology, The University of Texas Medical Branch at Galveston, Galveston, TX 77555, USA; 2 Galveston National Laboratory, The University of Texas Medical Branch at Galveston, Galveston, TX 77555, USA; 3 Department of Microbiology, Icahn School of Medicine at Mount Sinai, New York, NY 10029, USA; 4 Department of Pathology and Immunology, Washington University School of Medicine, St. Louis, MO 63110, USA; 5 Department of Microbiology & Immunology, The University of Texas Medical Branch at Galveston, Galveston, TX 77555, USA

**Keywords:** Ebolavirus, VP24, immune evasion, STAT1

## Abstract

Members of the Ebolavirus genus demonstrate a marked differences in pathogenicity in humans with Ebola (EBOV) being the most pathogenic, Bundibugyo (BDBV) less pathogenic, and Reston (RESTV) is not known to cause a disease in humans. The VP24 protein encoded by members of the Ebolavirus genus blocks type I interferon (IFN-I) signaling through interaction with host karyopherin alpha nuclear transporters, potentially contributing to virulence. Previously, we demonstrated that BDBV VP24 (bVP24) binds with lower affinities to karyopherin alpha proteins relative to EBOV VP24 (eVP24), and this correlated with a reduced inhibition in IFN-I signaling. We hypothesized that modification of eVP24-karyopherin alpha interface to make it similar to bVP24 would attenuate the ability to antagonize IFN-I response. We generated a panel of recombinant EBOVs containing single or combinations of point mutations in the eVP24-karyopherin alpha interface. Most of the viruses appeared to be attenuated in both IFN-I-competent 769-P and IFN-I-deficient Vero-E6 cells in the presence of IFNs. However, the R140A mutant grew at reduced levels even in the absence of IFNs in both cell lines, as well as in U3A STAT1 knockout cells. Both the R140A mutation and its combination with the N135A mutation greatly reduced the amounts of viral genomic RNA and mRNA suggesting that these mutations attenuate the virus in an IFN-I-independent attenuation. Additionally, we found that unlike eVP24, bVP24 does not inhibit interferon lambda 1 (IFN-λ1), interferon beta (IFN-β), and ISG15, which potentially explains the lower pathogenicity of BDBV relative to EBOV. Thus, the VP24 residues binding karyopherin alpha attenuates the virus by IFN-I-dependent and independent mechanisms.

## 1. Introduction

The genus Ebolavirus incudes Ebolavirus (EBOV), Bundibugyo virus (BDBV), Sudan virus (SUDV), Taï Forest virus (TAFV), Reston virus (RESTV), and the most recently discovered Bombali virus (BOMV) which differ widely in the pathogenicity in humans and non-human primates [1]. Among these, EBOV, SUDV, and BDBV have caused periodic outbreaks of human disease, and EBOV caused an epidemic in West Africa in 2013–2016 [2]. Severity of human disease caused by ebolaviruses varies greatly with the mean case fatality rates of 44% for EBOV, 34% for BDBV, 30% for SUDV, no documented fatalities for TAFV, and no severe disease for RESTV; pathogenicity of BOMV for human is unknown [2,3]. Although human infections can differ greatly in routes of infection, dose of the virus, and healthcare conditions, differences in virulence of individual ebolaviruses can be reliably compared in experimental infections of non-human primates, which demonstrate 100% fatality rates for EBOV and only 50–75% for BDBV (reviewed in Ref. [4]). Multiple mechanisms of immune evasion explain the high pathogenicity of ebolaviruses [5]. Two Ebolavirus proteins mediate interferon antagonism: eVP35 inhibits retinoic acid-inducible gene I (RIG-I)-like receptor signaling and type I interferon (IFN-I) induction [6,7,8,9,10], and eVP24 prevents IFN-induced Jak-STAT signaling [11,12,13,14]. Interestingly, in the closely related Marburg viruses, VP40 rather than VP24 inhibits IFN-I signaling. While wild-type (WT) Ebolaviruses do not cause a disease in rodents, rodent-adapted filoviruses do [4]. Mutational profiling of eVP35 and eVP24 have identified amino acids that are critical for virulence phenotypes in mice [15,16], guinea pigs [17,18], and also non-human primates [19].

eVP24 interaction with karyopherin alphas (KPNA) prevents translocation of tyrosine-phosphorylated STAT1 to the nucleus, resulting in the blockade of IFN-induced gene expression [14]. eVP24 expression also damages the nuclear envelope [20]. Comparison of the binding affinities of Ebolaviruses with different pathogenicity demonstrated that BDBV VP24 (bVP24) binds to KPNA weakly compared to both EBOV and RESTV VP24 (eVP24 or rVP24). Prior studies that defined the binding interface between eVP24 and KPNA also defined three distinct clusters of amino acids, with each displaying varying impact on the interaction. These findings, coupled with sequence comparison of these three Ebolaviruses identified the following four putative residues within cluster 1 (defined in Ref. [14]) in eVP24: P83, N135, R140, and V141. Importantly, these residues are located outside the proposed VP24 STAT-1 binding site [21] and therefore are unlikely to significantly affect binding to STAT1. These amino acids, when mutated to alanine or substituted with corresponding BDBV residues resulted in a reduced inhibition of IFN-I signaling [22]. In the meantime, multiple alanine substitutions were necessary to obtain near-complete loss of binding [14] suggesting that they do not have a significant effect on VP24 expression, folding, and stability. Collectively, these findings further highlight the significance of this interface to modulate the host responses. However, the significance of KPNA binding residues has not yet been defined in the context of infectious viruses. Therefore, the rationale of this study was to investigate the role of individual amino acids in the VP24 KPNA interface, including those which differ between EBOV and BDBV, in virus replication and inhibition of the IFN-I response in the context of infectious viruses. Using a panel of recombinant EBOVs with mutations in eVP24, we further define the impact of KPNA binding residues on EBOV replication. Our studies provide further mechanistic insights into how individual amino acids in eVP24 KPNA interface affect eVP24 functions, including those dependent on and independent of IFN-I. Our results further support the critical role of amino acids involved in binding to KPNA as a factor affecting Ebolavirus replication.

## 2. Materials and Methods

### 2.1. Cells

The Vero-E6 (African green monkey kidney epithelium) and 769-P (human kidney adenocarcinoma) cell lines were obtained from ATCC. The STAT1-deficient U3A cell line which is unresponsive to both IFN-α and IFN-γ [23] was obtained from George R. Stark (Lerner Research Institute, Cleveland, OH, USA). Huh7 cells were a generous gift from the Gordan lab at the University of California in San Francisco. Vero-E6 cells were maintained in Modified Eagle Medium (MEM), supplemented with 10% fetal bovine serum (FBS), 1% sodium pyruvate, 1% MEM non-essential amino acids (Sigma), and 0.1% gentamicin sulfate, and plated on tissue culture-treated 24-well plates. The 769-P cells were maintained in RPMI1640 medium, whereas U3A cells were maintained in DMEM and supplemented with 10% FBS and antibiotics. Huh7 cells were maintained in Dulbecco’s modified Eagle’s medium supplemented with 10% fetal bovine serum. All cell culture reagents were obtained from ThermoFisher Scientific, Waltham, MA, USA unless indicated otherwise.

### 2.2. Generation of Mutated Viruses

**Figure 1:**
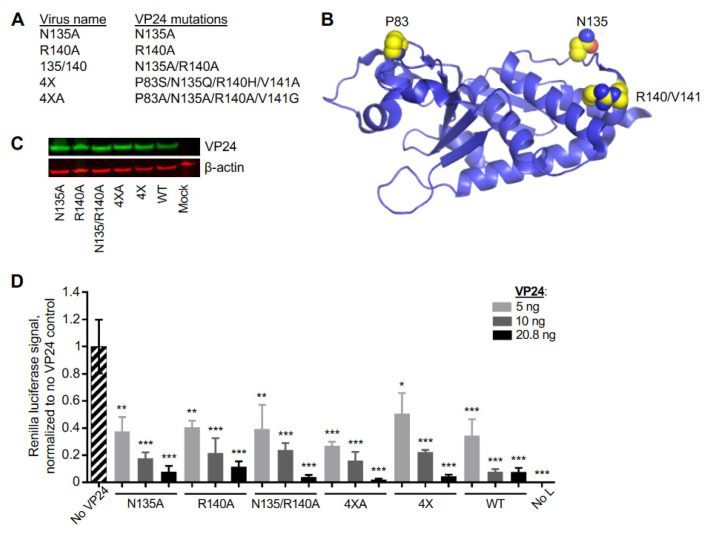
The EBOV VP24 mutants. (**A**). The virus names and the mutations. (**B**). Structure of VP24 displaying the mutated amino acids in yellow (PyMOL). (**C**). Western blot analysis of VP24 in lysates of Vero-E6 cells infected with EBOV-eGFP WT or its mutated derivatives. (**D**). EBOV tetracistronic minigenome reporter assay. Huh7 cells were transfected with the minigenome and support plasmids. The transfection also included the empty vector (No VP24) or plasmids expressing WT and mutated versions of VP24 at indicated amounts. On day 3, luciferase activity was quantified. Mean values based on triplicate samples ± SE. Statistical difference was assessed by one-way ANOVA–Dunnett’s multiple comparison test comparing the groups to no VP24 control: * *p* < 0.01, ** *p* < 0.001, *** *p* < 0.0001.

To generate the recombinant viruses with mutations in eVP24, we used the EBOV reverse genetics system which included the full length clone, representing the plasmid encoding the genomic RNA of WT EBOV under the control of T7 polymerase, previously modified by addition of the transcriptional cassette-encoding enhanced green fluorescent protein (eGFP) between the NP and VP35 genes [24], generously provided by Dr. J. Towner and Dr. S. Nichol (CDC). The system also included the five support plasmids encoding the EBOV proteins involved in replication and transcription (NP, VP35, L, VP30), and also T7 polymerase [25] kindly provided by Dr. Y. Kawaoka (University of Wisconsin) and Dr. H. Feldmann (NIH). The full-length clone was used to introduce the mutations shown in Figure 1. DNA fragments containing specific amino acid substitutions in eVP24 gene were synthesized by Integrated DNA Technologies (IDT, Coralville, IA, USA). The synthesized DNA fragments were digested with NcoI and SacI restriction endonucleases and cloned into a pUC19 subclone containing the SalI-SacI fragment of the pEBOV plasmid that included the VP24 gene. Subsequently, the pUC19 subclone containing the mutations were digested with SalI and SacI restriction endonucleases and cloned into the full-length clone. The recovered recombinant viruses were amplified by two passages in Vero-E6 cells, and the presence of the mutations was confirmed by sequencing the entire viral genomes; no reversions to the WT genotype or occurrence of any other mutations were observed in any part of the genome of any of the viruses.

The growth kinetics of the viruses were compared in triplicate Vero-E6 and 769-P monolayers; cells were infected at an MOI of 0.01 PFU/cell, incubated for 2 h, washed 3 times with 1× phosphate buffered saline (PBS), covered in MEM or RPMI1640 medium with 5% FBS for Vero-E6 and 769-P cells, respectively, and incubated for 6 days. To assess the growth kinetics in the presence of IFN-I, cells were treated with 100 U/mL of IFN-β (PBL Assay Science, Piscataway, NJ, USA) at four different timepoints, −6, 0, 24, and 48 h post-infection (hpi). eGFP fluorescence at different timepoints in the presence and absence of IFNs was photographed. Daily aliquots were collected, flash frozen and used for quantitation of the viruses by plaque titration. Plaques positive for eGFP were counted under a UV microscope. The viral growth was quantified using the area under the curve (AUC) using the multistep growth curve and calculated by the trapezoidal rule [26], using exact viral titers at 2–6 days post-infection, as determined by plaque assay in Vero-E6 and 769-P cells. 

### 2.3. EBOV Tetracistronic Minigenome Reporter Assay

Huh7 cells were seeded at a density of 3 × 10^4^ cells/well in a 96-well format. TransIt-LT1 transfection reagent (Mirus Bio) was used at a 3:1 ratio of reagent to DNA to transfect Huh7 cells with L at 166.7 ng, VP35 at 20.8 ng, NP at 20.8 ng, VP30 at 12.5 ng, T7 at 41.7 ng, and the EBOV tetracistronic minigenome at 41.7 ng per well. The EBOV tetracistronic minigenome encodes for Renilla luciferase, VP40, GP, and VP24 under a T7 promoter for initial transcription of the minigenome [27]. As a transfection control, 0.8 ng per well of firefly luciferase plasmid was included. In addition to the plasmids described above, VP24 WT or mutant plasmids were also transfected at 5, 10, or 20.8 ng per well, with empty pCAGGS vector as a control. At 72 h post-transfection, luciferase activity was measured using a dual-luciferase assay (Promega, Madison, WI, USA). Luciferase assay results were read using a BioTek Cytation 10 Confocal Imaging Reader (Agilent Technologies, Lexington, MA, USA).

### 2.4. ISG54 Luciferase Assay

Vero-E6 cells were transfected with an ISG54 firefly luciferase reporter plasmid and a constitutively active Renilla luciferase reporter plasmid (pRL-tk) (Promega). At 24 h post-transfection, the cells were infected with WT or the mutant viruses at an MOI of 2 PFU/cell. The infected cells were treated or not with 1000 U/mL of human IFN-β at either 24 or 48 hpi. At 24 h post-IFN-β treatment, cells were lysed, and a dual luciferase reporter assay (Promega) was performed. Firefly luciferase luminescence values were normalized to Renilla luciferase luminescence values. 

### 2.5. STAT1 Nuclear Translocation Assays

The assay was performed as described previously [11]. Briefly, Vero-E6 cells were seeded onto 8-well chamber slides and were infected with either WT or the mutant viruses expressing eGFP at an MOI of 2 PFU/cell. The infected cells were treated with 1000 U/mL human IFN-β for 30 min at 37 °C. Cells were washed twice with PBS containing 0.9 mM CaCl_2_ and 0.5 mM MgCl_2_ (PBS-CM), fixed with 4% paraformaldehyde for 30 min, permeabilized for 10 min with PBS containing 0.3% Triton X-100 and blocked for 45 min at room temperature with 4% normal goat serum in PBS containing 0.5% BSA, 0.15% glycine (PBG), and 0.3% Triton X-100. The fixed cells were incubated with rabbit anti-STAT1 (1:400) (5 mg/mL; Santa Cruz Biotechnology, Dallas, TX, USA) and Alexa Fluor 594 conjugated goat anti-rabbit IgG diluted 1:1000 in PBS containing 1% BSA and 0.3% Triton X-100 for 1 h at room temperature. Slides were washed 3 times with PBS-CM, fixed with 10% formalin for 48 h and removed from the BSL-4 containment. Slides were washed with PBS 3 times, incubated with 6-diamin-2-phenylindole-dihydrochloride (DAPI) (ThermoFisher Scientific) at the final concentration of 1 µg/mL for 3 min and washed 3 times in PBS. Slides were analyzed by laser scanning confocal microscopy using an Olympus FV1000 confocal microscope. For DAPI, a 405 nm laser and 425/50 filter were used, for eGFP, a 488 nm laser and a 515/30 filter were used, and for Alexa Fluor 594, a 543 nm laser and a 610/50 filter were used. All images were acquired using a 60× oil objective.

### 2.6. Viral Replication Kinetics in U3A Cells

U3A cells were plated on 12-well plates at 1 × 10^5^ cells per well and were infected with WT or mutant viruses at an MOI of 2 PFU/cell, adsorbed for one h, washed three times with PBS, covered in 1 mL of DMEM containing 5% FBS, and incubated for 2 days. eGFP fluorescence was photographed 48 h after infection. Supernatants were harvested, and the viruses were quantified by plaque titration in Vero-E6 cells as described above.

### 2.7. RNA Isolation

Both supernatants and cell lysates of infected cells were inactivated by adding Trizol (ThermoFisher Scientific) with subsequent incubation for 10 min at room temperature, and removed from the BSL-4 facility. RNA isolation was performed using a Direct-Zol RNA miniprep kit (Zymo Research, Irvine, CA, USA) according to the manufacturer’s recommendations, with on-column DNase treatment for cell lysates. The concentrations of the extracted RNA were measured on NanoDrop 2000 (ThermoFisher Scientific), and 200 ng was used in qPCR assays.

### 2.8. Quantification of gRNA in Supernatants

Viral titers in supernatants of infected Vero-E6 cells were measured by plaque assay as described above. EBOV gRNA in supernatants was measured by quantitative RT-PCR as previously described [28] with some modifications. First strand cDNA synthesis was performed with a negative-sense, nucleoprotein gene-specific primer and SuperScript IV reverse transcriptase (ThermoFisher Scientific) as per the manufacturer’s protocols. qRT-PCR was performed with the QuantStudio 6 Real-Time PCR system by combining 4 ng of cDNA, TaqManFast Advanced Master Mix, and custom primer-probe (FAM)-based qPCR assays (IDT). Absolute quantification was achieved using a standard curve generated by serial dilution of a virus stock template with known titer (PFU/mL).

### 2.9. Quantification of gRNA and mRNA in Cell Lysates

Strand-specific qRT-PCR assays to distinguish EBOV gRNA and VP35 mRNA were performed as previously described [29] using reverse transcription primers that contain unique 18–20 nucleotide tag besides the strand specific sequence. Absolute quantification was performed using amplicons purchased from IDT. 

### 2.10. Quantification of IFNλ1 Expression in 769-P Cells

cDNA synthesis was performed using oligo(dT)20 primers and SuperScript IV reverse transcriptase (ThermoFisher Scientific) as per the manufacturer’s instructions. The following qPCR primers were used to amplify IFNλ1: forward, GGAGCTAGCGAGCTTCAAGA; reverse, ACTCCAGTTTTTCAGCTTGAGTG; FAM probe, GCCAGGGACGCCTTGGAAGAG. Predesigned PrimeTime qPCR assays were purchased for ACTB control (IDT Assay ID: Hs.PT.39a.22214847). Multiplex qPCR assay was performed by combining primer-probes (IDT) for both the target gene (IFNλ1-FAM probe) and housekeeping gene (ACTB-Cy5 probe) in the same tube, and the relative expression level of the IFNλ1 gene was analyzed using the 2−ΔΔCT method [30]. gRNA quantitation in the 769-P cell lysates was also performed as described above and was used for the further normalization of IFNλ1 gene expression for each virus.

### 2.11. Quantification of IFNβ and ISG15 Expression in 769-P cells

A total of 0.2 µg of the RNA samples were reverse transcribed using the SuperScript™ IV First-Strand Synthesis System (ThermoFisher Scientific) based on the manufacturer’s protocol with appropriate primer pairs (2 µM). The oligonucleotide primer sequences used in the experiment are as follows—IFNβ F: GTCAGAGTGGAAATCCTAAG, IFNβ R: ACAGCATCTGCTGGTTGAAG, ISG15 F: TCCTGGTGAGGAATAACAAGGG, ISG15 R: GTCAGCCAGAACAGGTCGTC, β-actin F: ACTGGAACGGTGAAGGTGAC and β-actin R: GTGGACTTGGGAGAGGACTG. Real-time qPCR was conducted using PerfeCTa^®^ SYBR^®^ Green FastMix^®^ (QuantaBio, Beverly, MA, USA). The PCR reaction was performed on a Bio-Rad CFX Opus 96 qPCR instrument under the following conditions: 95 °C for 10 min, 40 cycles of 95 °C for 15 sec, 60 °C for 1 min. IFNβ and ISG15 were normalized using β-actin as the internal control and the fold difference in gene expression was calculated using the threshold cycle (ΔΔCT) method.

### 2.12. Analysis of VP24 by Western Blotting

Vero-E6 cells were infected with EBOV WT or mutant viruses at an MOI of 2 PFU/cell for 3 days at 37 °C, lysed in 4× SDS Laemmli Buffer, diluted to 1× in RIPA buffer, boiled for 15 min, and loaded on 4–12% gradient gels (all materials from ThermoFisher Scientific). Separated proteins were blotted onto nitrocellulose membranes using the I-Blot2 system (ThermoFisher Scientific), blocked for 1 h with Odyssey PBS blocking buffer and incubated with primary antibodies overnight at 4 °C. The following primary antibodies from ThermoFisher Scientific were used: rabbit polyclonal EBOV VP24 and mouse monoclonal actin antibody. Blots were washed 3 times for 5 min each with PBS buffer with 0.05% Tween-20 (PBST) on a shaker at room temperature. Anti-rabbit and anti-mouse fluorescent-conjugated secondary antibodies were purchased from LI-COR, Lincoln, NE, USA. Secondary antibodies were used at 1:15,000 dilution in PBST. Following 1 h-long incubation, blots were washed 3 times for 5 min each with PBST. Fluorescent bands were detected using an Odyssey Fc imaging system (LI-COR).

### 2.13. Statistical Methods

Statistical analyses were performed using GraphPad Prism version 6 (GraphPad Software). The following statistical tests were used: one-way ANOVA with multiple comparisons—Dunnett’s multiple comparison test (for the data shown in Figure 1 and Figure 5–8), two-way ANOVA with Tukey’s multiple comparison test (for the data shown in Figure 4), and two-way ANOVA with Dunnett’s multiple comparison test (for the data shown in Table 1).

## 3. Results

### 3.1. eVP24 KPNA Interface Amino Acids Are Required for Effective Replication of the Virus

eVP24 binds to the NPI-1 subfamily of KPNAs: KPNA1, KPNA5, and KPNA6 [11,13]. Interestingly, a previous study demonstrated that bVP24 binds to KPNA1, KPNA5, and KPNA6 with lower affinities compared to eVP24, resulting in a reduced inhibition of IFN-I signaling [11,12,13,14,21,22]. The amino acids on the eVP24-KPNA5 binding surface associated with this reduced binding affinity were identified: P83, N135, R140, and V141 [22]. To examine the role of these eVP24 amino acids in a context of EBOV infection, we designed a panel of five mutant viruses, each carrying either one or more amino acid mutations in VP24 and each expressing eGFP (Figure 1A,B). Specifically, we introduced point mutations N135A or R140A or both. We also designed two more recombinant viruses with substitutions in all four identified amino acids to that in bVP24 termed as eVP24 4× (eVP24 P83S/N135Q/R140H/V141A) or to alanine/glycine termed as eVP24 4 × A (eVP24 P83A/N135A/R140A/V141G). All five mutated viruses were successfully recovered and demonstrated no significant difference in eVP24 expression (Figure 1C). Over-expression of eVP24 can inhibit EBOV transcription [31]. To ensure that the mutations do not disrupt the overall folding and biological properties of the protein, we over-expressed the eVP24 constructs in a context of an EBOV tetracistronic minigenome assay that was developed based on a published system [27,32]. All of the VP24 mutants caused a dose-dependent reduction in the *Renilla* luciferase signal similar to WT VP24 (Figure 1D). Retention of this function suggests that the mutations do not disrupt the overall structure of VP24.

**Figure 2:**
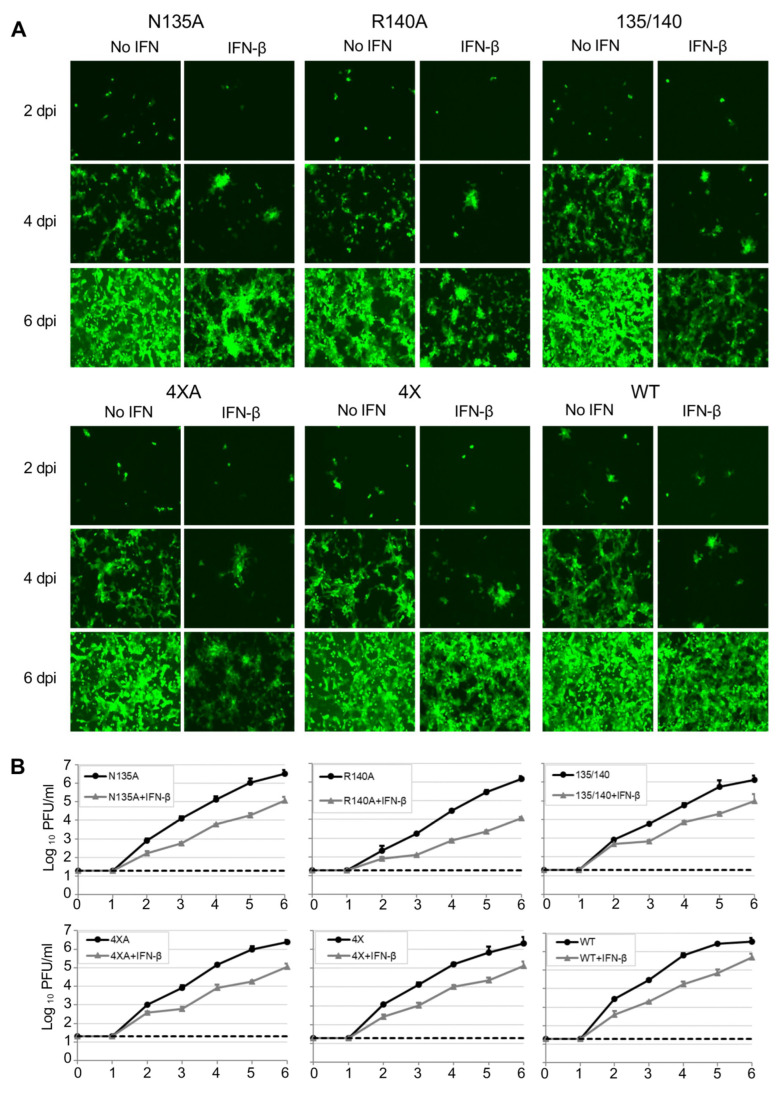
Characterization of the VP24 mutants in Vero-E6 cells. (**A**). The UV microscopy of cells infected with WT or mutant viruses and treated or not with IFN-β at 2, 4, and 6 dpi, 20× magnification. (**B**). Viral growth kinetics: titers in supernatants of Vero-E6 cells infected at MOl of 0.01 PFU/cell and treated or not with IFN-ß determined by plaque assay. Mean values based on triplicate samples ± SE.

**Figure 3:**
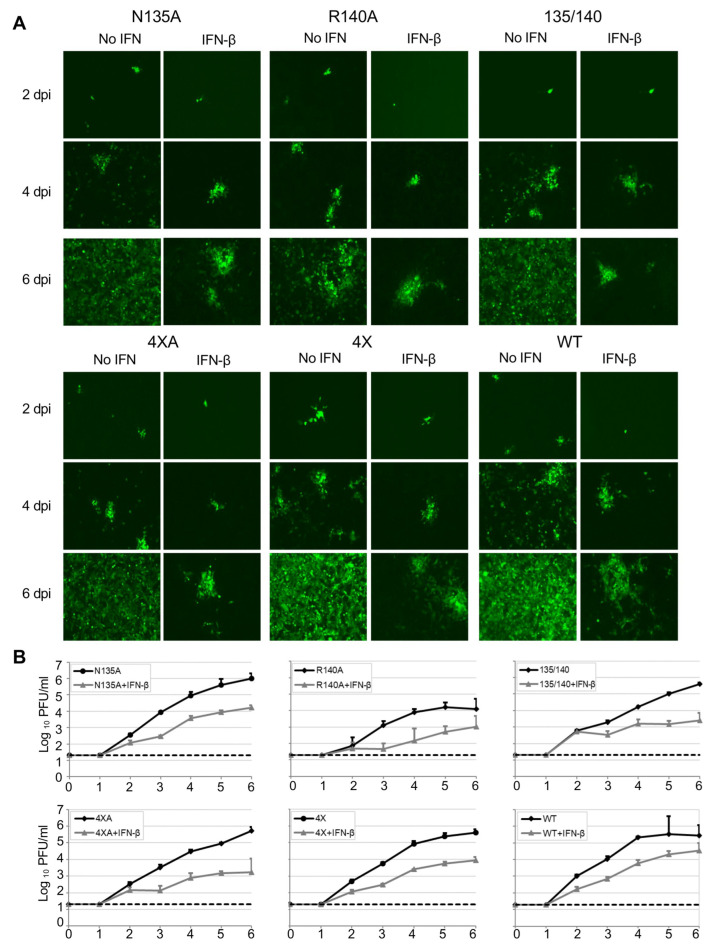
Characterization of the VP24 mutants in 769-P cells. (**A**). UV microscopy of cells infected with WT or mutant viruses and treated or not with IFN-β at 2, 4 and 6 dpi, 20× magnification. (**B**). Viral growth kinetics: titers in supernatants of Vero-E6 cells infected at MOl of 0.01 PFU/cell and treated or not with IFN-ß determined by plaque assay. Mean values based on triplicate samples ± SE.

As eVP24 antagonizes the IFN-I response, we next compared the multistep growth kinetics of these mutants both in Vero-E6 cells, which are deficient in IFN-I [33] and 769-P cells, which are IFN-I competent, either in the presence or absence of exogenously added IFN-β (Figure 2 and Figure 3, Table 1). Overall, the mutated viruses demonstrated decreased growth kinetics as compared to EBOV WT at 2–6 days post infection (dpi) in both cell lines with or without IFN-I, with the R140A mutant being the most attenuated (Table 1). Specifically, in Vero-E6 cells, a significant decrease in peak viral titer relative to WT virus was observed for all mutant viruses in the presence of IFN-I. Unexpectedly, the R140A and N135/R140A mutants appeared to be also attenuated in the absence of exogenously added IFN-I (Figure 2 and Table 1). In 769-P cells, a significant decrease in peak viral titer was observed for the R140A, N135/R140A, and 4 × A mutants in presence of added IFN-I, and the R140A mutant showed a significant decrease in the absence of added IFN-I (Figure 3 and Table 1). These results demonstrate that all the identified amino acids play roles in viral replication, with the most prominent role of R140 whose substitution resulted in a reduced viral growth in both cell lines even in the absence of added IFN-I.

### 3.2. eVP24 KPNA Interface Amino Acids Inhibit IFN-mediated STAT1 Nuclear Translocation

**Figure 4:**
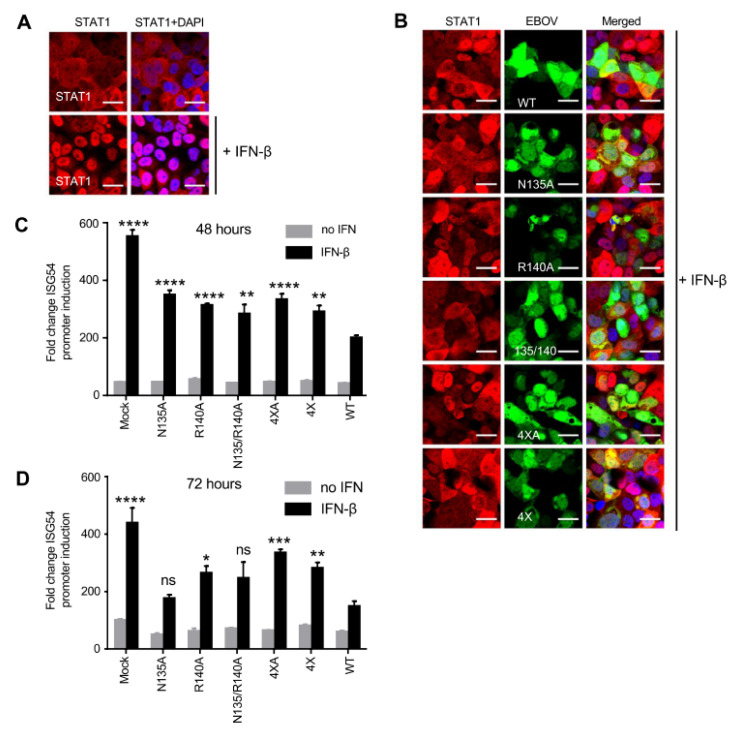
eVP24 mutants have reduced IFN-I inhibitory activity. (**A**,**B**). STAT1 nuclear translocation after 30 min-long IFN-βtreatment. (**A**). In uninfected Vero-E6 cells, STAT1 (red) translocates to the nucleus (DAPI staining, blue). Scale bar = 20 μm. (**B**). In infected Vero-E6 cells (green), STAT1 nuclear staining is reduced for EBOV-eGFP-WT (the top row) but increased for the mutant viruses. Scale bar = 20 μm. (**C**,**D**). ISG-54 reporter assay. Inhibition of IFN-induced ISG54 promoter activity in Vero-E6 cells at 48 h (**C**) and 72 h (**D**) after infection with EBOV-eGFP at MOI of 2 PFU/cell. Mean values based on triplicate samples ±SE. Statistical difference was assessed by two-way ANOVA—Tukey’s multiple comparison test comparing the mutants to WT EBOV: * *p* < 0.05, ** *p* < 0.01; *** *p* < 0.001; **** *p* < 0.0001; ^ns^ (not significant).

Previously, we demonstrated that eVP24 prevents the nuclear accumulation of STAT1 thereby preventing IFN-induced gene expression [11]. To determine the role of these eVP24 mutants on STAT1 translocation to the nucleus, we infected Vero-E6 cells with WT or mutant viruses at MOI of 2 PFU/cell, incubated cells for 48 h, added IFN-β at 1000 U/mL, incubated the cells for an additional 30 min, and fixed and stained cells (Figure 4A,B). As expected, infection with EBOV WT almost completely prevented STAT1 nuclear localization, whereas infection with the mutants resulted in detectable STAT1 nuclear localization.

Previously, using a plasmid-based system, we demonstrated that two of the eVP24 mutants, 4 × A and 4×, did not inhibit the ISG54 reporter activity as effectively as EBOV WT [22]. To determine how the two eVP24 mutants affect IFN-I induction, cells were transfected with an ISG54 reporter plasmid and infected with EBOV WT or the mutant viruses at an MOI of 2 PFU/cell. Twenty-four or 48 hpi cells were treated with IFN-β at 1000 U/mL or not treated for 24 h, and the reporter activity was assessed. As expected, EBOV WT caused a significant decrease in ISG54 reporter activity compared to mock-infected cells at both time points. Importantly, we observed a significant decrease in IFN-I inhibiting activity for all mutant viruses compared to EBOV WT at 48 hpi (Figure 4C). This significant reduction in inhibiting activity was also observed at 72 hpi for the R140A, 4× and 4 × A mutant viruses (Figure 4D). These results suggest that the reduced growth kinetics of these mutant viruses in the presence of IFN-β is due to their inability to effectively block IFN-I signaling.

### 3.3. eVP24 R140 Is Required for Effective EBOV Replication Independent of IFN-I Antagonism

**Figure 5:**
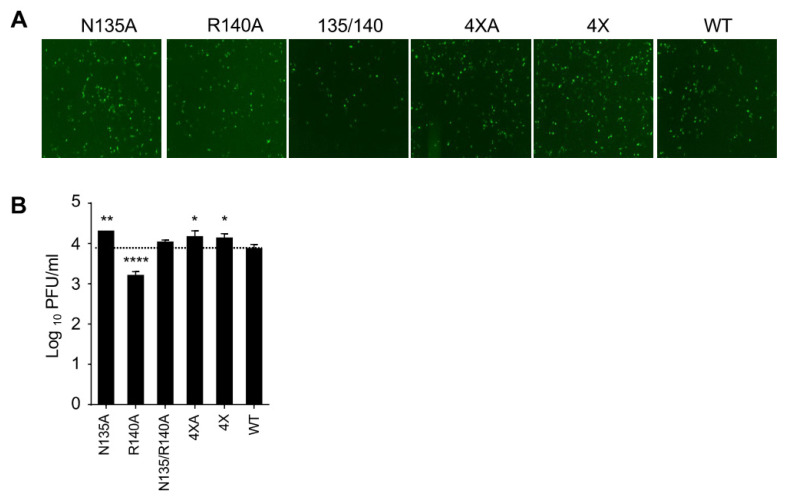
The R140A mutation in eVP24 inhibits viral growth in U3A (STAT1-KO) cells. Cells were infected with the indicated viruses at MOl of 2 PFU/cell and analyzed at 48 hpi. (**A**). UV microscopy, 20× magnification. (**B**). Viral titers in the supernatants. Mean values based on triplicate samples ± SE. Statistical difference was assessed by one-way ANOVA–Dunnett’s multiple comparison test comparing the mutants ‘ to WT EBOV: * *p* < 0.05, ** *p* < 0.01, **** *p* < 0.0001.

To determine the impact of STAT signaling on EBOV replication, we determined the growth kinetics of these mutant viruses in U3A cells, a human cell line deficient in STAT1 [23]. Many viruses replicate well in STAT1 deficient cell lines due to deficient IFN responsiveness [34,35]. Although EBOV was able to replicate in U3A cells, the titers were lower than in Vero-E6 cells. We did not observe decreased viral titers for four of the mutant viruses at 2 dpi relative to WT (Figure 5A,B). The R140A mutation, however, led to a significant decrease in the viral titer compared to WT virus (Figure 5A,B). The reduced growth of the R140A mutant in STAT1 deficient cells suggests that the R140A mutation at least in part acts via an IFN-I-independent pathway, while the growth phenotypes of the remaining mutant viruses appear to be IFN-I-dependent. These data suggest that R140, despite being located in the KPNA interface, is required for effective viral replication at least in part not due to IFN-I antagonism.

### 3.4. Mutations in eVP24 KPNA Interface Reduce the Amounts of Both Viral Genome and mRNA

**Figure 6:**
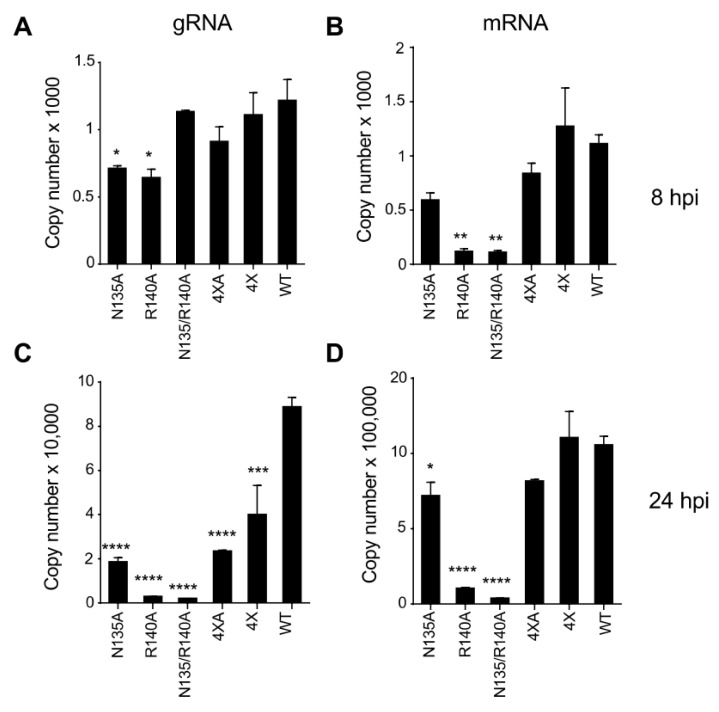
Effect of VP24 mutations on the amounts of EBOV genomic RNA and mRNA. Vero-E6 cells were infected with the panel of viruses at MOl of 2 PFU/cell, and the cells were harvested at 8 hpi (**A**,**B**) or 24 hpi (**C**,**D**) and analyzed by qRT-PCR for viral genomic RNA (gRNA) (**A**,**C**) or VP35 mRNA (**B**,**D**). The values show the mean numbers of copies in a 2 ng reaction based on triplicate samples ± SE. Statistical differences were assessed by one-way ANOVA–Dunnett’s multiple comparison test comparing the mutants to WT EBOV: * *p* < 0.05. ** *p* < 0.01, *** *p* < 0.001; **** *p* < 0.0001.

eVP24 has been known to regulate both replication and transcription of the EBOV genome [31,36], but at present the role of the KPNA interface in replication and transcription has yet to be described. We used the panel of eVP24 mutated viruses to compare the amounts of their genome and mRNA to WT virus. We infected Vero-E6 cells with EBOV WT or the mutant viruses at an MOI of 2 PFU/cell, harvested cells at 8 and 24 hpi, and performed strand-specific qRT-PCR for both viral genomic RNA (gRNA) and mRNA (Figure 6). Two single mutants, N135A and R140A, demonstrated a decrease in gRNA levels at 8 hpi (Figure 6A), while all five mutants demonstrated significantly reduced gRNA relative to WT at 24 hpi (Figure 6C).

Further, the R140A mutant and the double mutant N135/R140A demonstrated significantly reduced amounts of mRNA at 8 hpi (Figure 6B), and three mutants, N135A, R140A, and the double mutant N135/R140A demonstrated significantly reduced amounts of mRNA at 24 hpi (Figure 6D). The reduction in both gRNA/mRNA for the mutants N135A and R140A support their role in genome replication and/or transcription. It has been shown that eVP24 plays a role in the regulation of viral replication and transcription [31,36,37], and therefore the residues N135 and R140 are likely to be involved in these processes. Thus, the reduced growth of the R140A virus in STAT1 KO cells (Figure 5B) can be attributed to the involvement of R140 in an as yet poorly defined process that involves EBOV genome replication and/or transcription.

**Figure 7:**
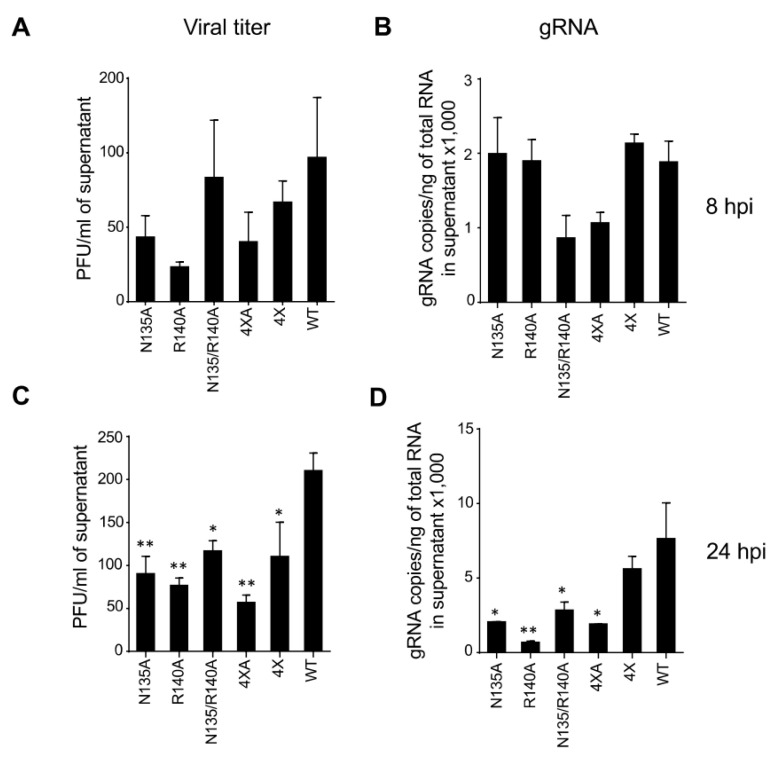
Effect of VP24 mutations on EBOV replication. Vero-E6 cells were infected with the panel of viruses at MOI of 2 PFU/cell, the supernatants were collected at 8 hpi (**A**,**B**) or 24 hpi (**C**,**D**), and the viral titers were determined by plaque assay (**A**,**C**) and qRT-PCR (**B**,**D**). Mean values ± SE based on triplicate samples. Statistical differences were assessed by one-way ANOVA–Dunnett’s multiple comparison test comparing the mutants to WT EBOV: * *p* < 0.05; ** *p* < 0.01.

Next, we compared the amounts of viral particles released in the media by measuring viral titers by plaque assay, as well as quantifying gRNA by qRT-PCR. At 8 hpi, some of the mutants demonstrated reduced titers which appeared not to be significant due to the high sample-to-sample variation (Figure 7A,B). However, at 24 hpi, a significant reduction in viral titers (57%, 63%, 44%, and 73% for N135A, R140A, N135/R140A, and 4 × A mutants, respectively) was observed in both assays for all mutants except the 4× mutant relative to WT (Figure 7C,D). For the 4× mutant, which has all four mutated residues analogous to BDBV, a modest but statistically significant reduction of 48% was observed only by the plaque assay. These results demonstrate the involvement of all four amino acids in EBOV replication.

### 3.5. Unlike WT eVP24, eVP24 with bVP24 Interface Residues Does Not Inhibit IFNλ1 or IFNβ Expression

**Figure 8:**
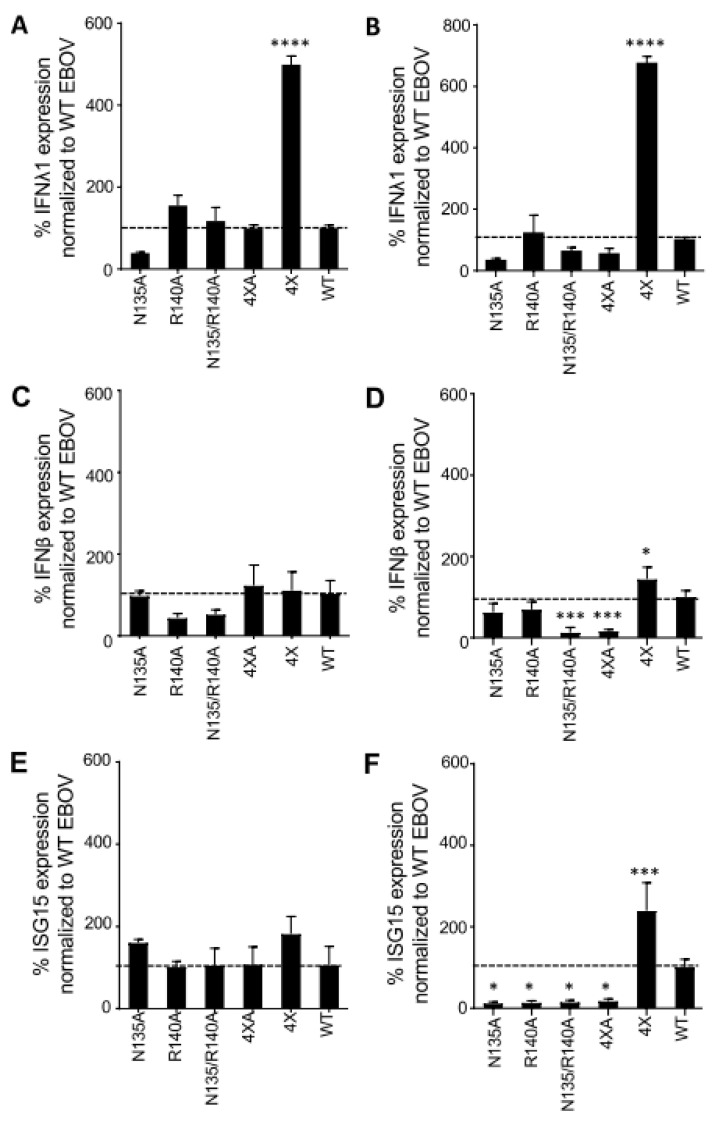
Effect of mutants on IFNA1, IFNβ, ISG15 expression. Gene expression—IFNA1 (**A**,**B**), IFNβ (**C**,**D**), and ISG15 (**E**,**F**)—was quantified by qRT-PCR in 769-P cell lysates infected with EBOV-eGFP at MOl of 2 PFU/cell after 2 dpi (**A**,**C**,**E**) and 3 dpi (**B**,**D**,**F**). Gene expression was normalized to gRNA and expressed relative to WT (100%, shown with the dotted line). Statistical differences were assessed by one-way ANOVA—Dunnett’s multiple comparison test comparing the mutants to WT EBOV: **** *p* < 0.0001; *** *p* < 0.001; and * *p* < 0.05.

EBOV infection is characterized by a very effective inhibition of IFN-I (IFNα, IFNβ) production and signaling, and both eVP35 and eVP24 viral proteins play a critical role in this inhibition [38]. Recently, eVP24 has also been shown to inhibit IFNλ1 gene expression, and this inhibition was lost when KPNA binding residues were mutated [39]. To determine if any of the mutations in this study affect the induction of IFNλ response, we measured IFNλ1 in 769-P cells infected with EBOV WT or the mutant viruses by qRT-PCR. Strikingly, we observed a 5.5- and 6.7-fold increase in IFNλ1 level at 2 and 3 dpi, respectively, for the 4× mutant which has all four residues identical to that of BDBV, and no increase for any of the other four mutants (Figure 8A,B). Our data suggest that these four residues on eVP24 are critical for its IFNλ antagonistic activity. 

To determine whether we see a similar expression pattern for type I IFN in these mutant viruses, we quantified the gene expression of both IFN-β, as well as ISG15 which is induced by type I IFN. For both targets, we did not observe any differences for any of the mutants relative to WT at 2 dpi (Figure 8C,E). However, at 3 dpi we observed a significant decrease in both IFN-β and ISG15 levels for two mutants—N135/R140A and 4 × A (Figure 8D,F). Additionally, the two single mutants—N135A and R40A showed a decrease in ISG15 expression only. Conversely, the 4× mutant showed a significant increase in both I IFN-β and ISG15 expression. Thus, the 4× mutant causes an increase in both type-I and type III IFN response. Taken together, these data suggest a novel role for the KPNA interacting cluster 1 residues and a possible mechanism of the lower pathogenicity of BDBV as compared to EBOV.

## 4. Discussion

We performed this study to determine if the KPNA binding amino acids in bVP24 can alter EBOV infection. Incorporation of the selected mutations in the viral genome and successful recovery of infectious, mutated viruses suggest that the mutations did not significantly affect VP24-specific functions. We observed a significant attenuation of all five mutated viruses in the presence of exogenous IFN-I in Vero-E6 cells, but only three of the four alanine mutants were attenuated in IFN-competent 769-P cells. Similarly, all five mutant viruses inhibited IFN-induced ISG54 gene expression less efficiently than WT EBOV due to increased STAT1 nuclear translocation. Additionally, only the EBOV mutant containing all four amino acids identical to that in BDBV caused a significant increase in IFNλ1, IFNβ, and ISG15 gene expression. Furthermore, the mutant R140A was significantly attenuated in STAT1-KO cells and demonstrated a significant reduction in the amounts of viral gRNA and mRNA. Thus, the VP24 residues binding KPNAs are likely to be responsible for the reduced replication of BDBV compared to EBOV and may influence Ebolavirus pathogenicity both by IFN-I-dependent and IFN-I-independent mechanisms. 

We observed reduced growth kinetics for all eVP24 mutant viruses in Vero-E6 cells, mainly in the presence of IFN-I. Most importantly, one of the mutants, R140A, was severely attenuated in both Vero-E6 and 769-P cells, irrespective of the presence or absence of exogenous IFN-I, suggesting a critical role for this amino acid in viral pathogenesis in an IFN-I-independent manner. The paradoxical lack of attenuation in the presence of additional mutations suggests possible compensatory effects. 

It was demonstrated that the eVP24 residues that bind to karyopherin alpha are necessary for IFN-I antagonistic activity in a plasmid-based transfection system [22]. Moreover, one study provided evidence that eVP24 can also directly bind STAT1 and identified residues on VP24 responsible for this interaction [21]. In the current study, we demonstrated that all five mutated viruses, generated based on these structural studies and prior comparison of eVP24 and bVP24, did not block STAT1 translocation to the nucleus resulting in enhanced expression of IFN-stimulated genes. To delineate STAT1-independent effects, we also examined the growth of these viruses in U3A cells, a STAT1-deficient cell line. The single mutants N135A and R140A showed opposite effects, while the double mutant demonstrated no change compared to the WT virus. Mutations in the IFN-I antagonist C protein of the Sendai virus led to reduced growth kinetics in IFN-I-responsive 2fTGH cells but similar growth in STAT1-deficient U3A cells suggesting that the IFN-inhibiting capacity of the C protein was critical for effective replication [34]. In this study, only the R140A mutant, which was attenuated in STAT1-deficient U3A cells, was also attenuated in IFN-I-responsive 769-P cells, suggesting a STAT1- and IFN-I-independent mechanism of attenuation.

It is interesting that the Ebolavirus VP24 residue 140, which is located in the cluster 1 of the KPNA binding residues, is not conserved between EBOV, BDBV, and RESTV [21]. The location of the residue adjacent to the KPNA1 binding domain of VP24 that resides in the amino acid 142–146 loop [12] led to speculation that it is responsible for the different pathogenicity of the three Ebolaviruses [40,41]. Additionally, the VP24 residues 137 and 140 are also critical for KPNA5 binding and had the greatest impact on IFN-I-sensitive response element activity [14]. Multiple residues in this region have been identified as specificity determining positions (SDPs) which are positions that are conserved within protein subfamilies but differ between them. It was suggested that SDPs distinguish between the different functional specificities of proteins from the different Ebolavirus species based on differences between the pathogenic and non-pathogenic Ebolaviruses by molecular dynamics and structural analysis [40,42,43]. However, this is the first study besides the one with the K142A mutant [44] wherein multiple eVP24 residues have been mutated and characterized in context of recombinant viruses.

Unlike eVP35 that plays a major role in both IFN antagonism and viral transcription, the role of the eVP24 in viral transcription is less clear. Initial studies with plasmid-based monocistronic minigenome systems showed that eVP24 inhibits both genome replication and transcription [31,36]. However, later studies using a tetracistronic minigenome, as well as infectious EBOV, demonstrated that the inhibiting effect of eVP24 on genome replication and transcription is modest [32]. Recently, it has been demonstrated that residues in the YxxL motif (amino acids 172–175) are critical for this inhibitory effect of eVP24 on transcription and replication [45]. The current study addressed the effects of eVP24, with single and combined mutations N135A and R140A in a region outside the YxxL region, that caused a significant reduction in the amounts of gRNA and mRNA relative to WT. As the R140A mutant demonstrated a reduced growth in STAT1-deficient U3A cells, the attenuation is likely to be attributed to the role of R140 in viral transcription and/or replication. Although none of the mutations significantly affected the VP24 expression and had no impact on the capacity of eVP24 to inhibit transcription of a tetracistronic minigenome, we cannot completely rule out that these mutations could have subtle effects on protein folding.

Recent studies have demonstrated that the binding of eVP24 to KPNA is also critical for the inhibition of IFNλ1 gene expression [39]. They confirmed that the mutant L201A/E203A/P204A/D205A/S207A, which did not bind KPNA [14], was unable to inhibit IFNλ1 gene expression. In our study, we evaluated other KPNA binding residues and found that only the 4× mutant (P83S/N135Q/R140H/V141A), which had BDBV residues at all four positions, induced a significantly increased expression of IFN-λ1 as compared to the WT virus. Furthermore, only this 4× mutant caused a significant induction of both IFN-β as well as ISG15. These results support prior studies wherein the mutation of KPNA binding sites in eVP24 reduces nuclear localization thereby reducing its ability to efficiently inhibit IFN gene expression [14,39,46]. Thus, the reduced inhibition of both IFN-I and IFN-III activity may contribute to the reduced pathogenicity of BDBV relative to EBOV.

The limitations of the study include that, at least theoretically, the mutations in eVP24-karyopherin alpha interface may affect viral entry and uncoating. However, given the lack of any significant effect on VP24 conformation (Figure 1B,C) and transcription inhibition function (Figure 1D), we believe this scenario is unlikely.

Taken together, our studies for the first time evaluate the effect of a key KPNA binding interface of VP24 with infectious recombinant viruses. More importantly, our studies identify IFN-I-dependent and IFN-I-independent effects of eVP24, including virus replication. Future studies are required to fully define the mechanisms by which the KPNA binding interface impacts virus replication, to investigate if these mutated viruses are attenuated in animal models and to determine if they can induce a protective immune response. Collectively, these studies further highlight the significant role played by the KPNA binding interface in a key virulence factor and a critical therapeutic target, eVP24.

## Figures and Tables

**Table 1:** Growth of the mutated viruses in Vero-E6 and 769-P cells in the presence and absence of exogenously added IFN-β.

	Vero-E6 Cells		769-P Cells	
Virus	No IFN-β Added	IFN-β Added	No IFN-β Added	IFN-β Added
N135A	19.98 (6.5 ^ns^)	14.48 (5.1 ****)	18.78 (6 ^ns^)	13.12 (4.2 ^ns^)
R140A	17.42 (6.2 *)	11.32 (4.1 ****)	14.18 (4.1 ****)	8.9 (3.0 ****)
135/140	18.83 (6.1 *)	14.85 (5.0 ****)	16.72 (5.6 ^ns^)	11.97 (3.4 ***)
4 × A	19.8 (6.4 ^ns^)	14.82 (5.1 ****)	17.07 (5.7 ^ns^)	10.9 (3.2 ****)
4×	19.85 (6.3 ^ns^)	15.18 (5.1 ****)	18.17 (5.6 ^ns^)	12.6 (3.9 ^ns^)
WT	21.68 (6.5)	16.52 (5.7)	19.12 (5.4)	14.28 (4.5)

Area under the curve (AUC) and mean peak viral titers in log_10_ PFU/mL (in brackets) of mutated and WT viruses in Vero-E6 and 769-P cells treated or not with IFN-β. Statistical significance of the difference in titers for the mutant viruses relative to WT was calculated by two-way ANOVA—Dunnett’s multiple comparison test. **** *p* < 0.0001; *** *p* < 0.001; * *p* < 0.05; ^ns^ (not significant).

## Data Availability

Not applicable.
